# Diversity of endophytic bacteria isolated from leguminous agroforestry trees in western Kenya

**DOI:** 10.1186/s13568-024-01676-6

**Published:** 2024-02-08

**Authors:** William Omuketi Emitaro, Fanuel Kawaka, David Mutisia Musyimi, Asenath Adienge

**Affiliations:** 1 Department of Biological Sciences, Jaramogi Oginga Odinga University of Science and Technology, 210, Bondo, 40601 Kenya; 2 Department of Botany, Maseno University, Private Bag, Maseno, Kenya; 3 Department of Biotechnology, Kenya Forestry Research Institute, 20412-00200, Nairobi, Kenya

**Keywords:** Niches, Morphological, Characterization, Molecular, Spherical, Rod shaped

## Abstract

Plants have diverse and vast niches colonized by endophytic microorganisms that promote the wellbeing of host plant. These microbes inhabit internal plant tissues with no signs of ill health. Bacterial endophytes from many plants have been isolated and characterized due to their beneficial roles however their diversity in leguminous plants still remain unexploited. Diversity of bacterial endophytes isolated from *Sesbania sesban*, *Leucaena diversifolia* and *Calliandra calothyrsus* was assessed using morphological and molecular characteristics. A total of 27 pure isolates were recovered from *C. Calothyrsus, L. diversifolia* and *S. sesban* constituting 44.4%, 33.3% and 22.2% from the leaves, stems and roots respectively. The isolates differentiated into Gram positive and negative with rods and spherical shapes. Analysis of 16S rRNA gene sequences revealed 8 closely related bacterial genera that consisted of *Bacillus* (33.3%), *Staphylococcus* (22.2%), *Alcaligens* (11.1%)*, Pantoea* (11.1%), *Xanthomonas,*and *Sphingomonas* (7.4%) each. Others included *Acinetobacter,* and *Pseudomonas* at 3.7% each. Bacterial endophytes of genus bacillus were isolated from all the three plants. These results indicate the presence of high diversity of endophytic bacteria associated with the different parts of *L. diversifolia, S. sesban* and *C. salothyrsus* growing in western Kenya.

## Introduction

Plant microbe interaction has been the subject of interest in current research due to its mutuality and biotechnological applications. Plants have diverse and vast niches which are colonized by microbes called endophytes that promote plant development and plant health (Bhagya et al. 2019; Emitaro et al. 2020). Endophytes are heterotrophic microorganisms inhabiting the inner plant environment with no sign of ill health (Li et al. 2020; Niem et al. 2020). Endophytes comprise of bacteria, fungi and actinomycetes distributed in every tissue, organ and plant species worldwide (Anyasi and Atagana 2019). Endophytes get into different plant tissues via germinating radicals, natural openings such as stomata and secondary roots. They may also gain entry through mechanically damaged foliar or by use of hydrolytic enzymes they secrete to degrade cell wall that acts as a barrier to advancing microbial pathogen (Dashyal et al. 2019; El-Deeb et al. 2013). Once inside the host plant, they may colonize the point of entry or may translocate to new sites and colonize intracellular or extracellular spaces of different parts of the plant parts to establish a mutual relationship with the plant (Coêlho et al. 2011; Khare et al. 2018; Suman et al. 2016).

In the recent past, endophytes have received wide attention due to their protective and growth enhancement roles in host plants (Abdennabi et al. 2017; Selim et al. 2016). They have shown unique intrinsic lifestyles and mechanisms to evade host defence reactions and bypass the host immune system to enable asymptomatic proliferation within the host (Basumatary et al. 2021). Reports by Sinha et al. (2017) and Tidke et al. (2017) show that endophytes can synthesize secondary metabolites such as peptides, quinolons, polyketones,alkaloids, phenolic compounds, steroids, flavonoids, terpenoids, azadirachtin and siderophores that have antimicrobial and insecticidal properties. Similarly, endophytes have unique enzyme systems that are responsible for synthesizing enzymes such as amylases, pectinases, laccases, cellulases, proteinases and lipases that arrest the proliferation and attack by microbial and insect pathogens (Fouda et al. 2015; Handayani et al. 2018).

Endophytic bacteria establish a beneficial relationship with host plants after entry by being protected from adverse environmental conditions while promoting growth and tolerance of the plant to stresses due to abiotic and biotic factors (Bind and Nema 2019; Brígido et al. 2019). Bacterial endophytes improve health and growth of the host plant through solubilization of phosphates, synthesis of phytohormones, production of siderophores and enhancement of nitrogen fixation (Gamalero et al. 2020). Moreover, endophytic bacteria exhibit antimicrobial properties that protect host plants from pathogenic microorganisms and their metabolites have been integrated into various biotechnological applications (ALKahtani et al. 2020; Mahadevamurthy et al. 2016). Due to the beneficial roles played by bacterial endophytes in their host plants, they have been isolated and characterized (Chowdhary and Kaushik, 2015; Mahadevamurthy et al. 2016) from different plants including non-leguminous and leguminous plants but there are still many plants whose endophytes have not been identified. In leguminous plants, endophytic bacteria are dominated by *Bacillus, Pseudomonas, Burkholderia, Rhizobium*, and *Klebsiella* (Brígido et al. 2019; Webster et al. 2020). Even though bacterial endophytes from some leguminous plants have been characterized *(*Brígido et al. 2019; Domka et al. 2019; Rozpądek et al. 2018; Webster et al. 2020), more studies are still required to understand bacterial endophytes associated with *Sesbania sesban*, *Leucaena diversifolia* and *Calliandra calothyrsus*. The three plants are economically important to small holder farmers as they are intercropped with food crops to enrich soils by fixing nitrogen and used as fodder for animals. Knowledge of the bacterial endophytes colonizing these plants would be of great interest in understanding their role and application in crop production besides being used for nitrogen fixation. The present study assessed the diversity of endophytic bacteria colonizing *Sesbania sesban, Leucaena diversifolia* and *Calliandra calothyrsus* growing in western Kenya.

## Materials and methods

### Study site, sampling and processing

Plant parts including roots, leaves and stems of *S. sesban, C. diversifolia* and *C. calothyrsus* were obtained separately from Maseno University farm in khaki bags. The University is located along Kisumu Busia road and lies at 0° 10ʹ 0″ South, 34° 36ʹ 0″ East. Plant materials collected were taken to the Microbiology laboratory of Jaramogi Oginga Odinga University of Science and Technology for processing. Plant materials were obtained in triplicates from demonstration plots and eventually pooled together before the isolation of endophytic bacteria.

### Isolation of bacterial endophytes

Plant roots, leaves and stems were first washed in running tap water to remove any soil or contaminant from the field before being washed in 70% ethanol for 5 min. They were transferred to 3% sodium hypochlorite for five minutes for complete surface sterilization and then rinsed several times in sterile distilled water (Yousefi et al. 2018). The efficiency of surface sterilization was assessed by inoculating 100 µL aliquot of the last rinsing water on Nutrient agar plates and incubating for 48 h at 28 ± 2 ℃. Absence of any growth indicated complete surface sterilization. Surface sterilized plant parts were crushed in 5 ml distilled water and one milliliter serially diluted up to 10^–4^. Bacteria endophytes were isolated on nutrient agar using the pour plate method for each plant species and plant part. Triplicate plates were incubated for 48 h at 28 ℃ arranged in a completely randomized design. Colonies emerging from the plates were subcultured separately 2–3 times based on morphological differences to obtain pure cultures.

### Morphological characterization of endophytic bacteria

Bacterial endophytes were characterized using colony characteristics such as colour, cell shape and arrangement, type of edge, opacity and appearance of cells after Gram staining (Thanh and Diep 2014). The shape of the cell and Gram's reaction were determined by observation under a light microscope (Leica DM 500) at × 100 (Prasad and Dagar 2014).

### Molecular characterization

#### Genomic DNA extraction

Zymo Research DNA Mini Prep^™^ kit(ZR, South Africa) was used for DNA extraction. Nanodrop^™^ Lite Spectrophotometer (Thermo Scientific Inc, USA) was used to estimate the concentration of DNA at 260–280 nm wavelengths. Horizontal gel electrophoresis (Thistle Scientific Ltd, USA) was used to estimate the purity on a 1% (w/v) agarose gel at 100 V for 40 min. The gel was stained with SYBR Safe dye (Invitrogen 10,000 × concentrate in DMSO) and visualized under UV (Adienge et al. 2019).

### 16S rRNA gene amplification

The identification of the bacterial endophyte isolates by 16S rRNA gene partial sequencing was performed using universal primers 1492R (5ʹTACCTTGTTACGACTT-3ʹ) and 27F (5ʹAGAGTTTGATYMTGGCTCAG-3ʹ) (Bind and Nema 2019). Amplification was carried out in a 20 μL PCR tubes each containing 1.4 μl Mgcl_2_, 2 μl DNA, 2 μl Taq buffer, Taq DNA Polymerase 0.4 μl, dNTPs 0.4 μl, Primers 2 μl and Nuclease free water 11.8 μl. The mixtures were transferred to a 96 well thermocycler (Applied Biosystems).Thermocycler was optimized to run at the following temperatures; initial denaturation for 5 min at 94 ℃, denaturation for 30 s at 94 ℃, annealing for 30 s at 47 ℃, elongation at 72 ℃ for 2 min and a final elongation for 10 min at 72 ℃. The cycles for denaturation, annealing and elongation were repeated 35 times. Products of amplification were separated on 2% (w/v) agarose gel in 1X TAE buffer, stained with SYBR Safe dye (Invitrogen 10,000 × concentrate in DMSO),and visualized under UV illumination table (ATTA E-Graph).

### DNA sequencing and phylogenetic analysis

The PCR products recovered were sent to Macrogen Europe B.V. (Meibergdreef 311,105 AZ, Amsterdam, Netherlands) for sequencing. Forward and reverse gene sequences obtained were imported to Geneious Prime^®^ 2020.0.4 (www.geneious.com) and contigs with approximately 1000 bp generated through De Novo assembly. Sequences were analyzed using BLASTn tool at the National Centre for Biotechnology Information database (NCBI) GenBank using the Basic Local Alignment Search Tool (BLAST) (http://blast.ncbi.nlm.nih.gov/Blast.cgi) (Bind and Nema 2019). A similarity search of 16S rRNA sequences was performed to identify closely related sequences available in the GenBank. Assembled multiple sequences greater than 1000 bp were transferred to MEGA Version 6.0 software and aligned using Clastal W method (Tamura et al. 2011). Sequences with greater similarity and from plant bacteria were retrieved for phylogenetic analysis. Evolutionary histories and diversity of the isolates were determined using the Neighbour-Joining method and distances computed using Maximum Composite Likelihood (Tamura et al. 2011). A bootstrap test (1000 replicates) was used to cluster associated taxa and replicate trees with above 50% likelihoods indicated on the branches. Endophytic fungi Penicillin was used as outgroup.

## Results

A total of 27 different colonies of bacteria were isolated from leaves, stems and roots of the three agroforestry trees. More bacterial endophytes were recovered from the leaves compared to the stems while roots had the least percentage recovery (Table 1). Most bacteria were recovered from *C. calothyrsus* followed by *S. sesban.*

**Table 1:** Total and Percentage bacterial endophytes recovered from roots, stems and leaves, of *L. diversifolia, C. calothyrsus* and *S. sesban*

Plant species	Bacterial recovery per plant part			
	leaf	Stem	root	Total
*C. calothyrsus*	5	4	3	12
*L. diversifolia*	3	2	1	6
*S. sesban*	4	3	2	9
Total	12	9	6	27
% Recovery	44.4	33.3	22.2	

### Phenotypic characterization of the bacterial isolates

Based on phenotypic characteristics, the isolates were characterized using colony appearance, morphology such as elevation, type of margin, opacity and appearance after Gram staining (Table 2). Yellow raised colonies with entire margins, opaque, cocci in cell shape and Gram negative were recovered from all the three plant parts. White colonies lying flat on the media with an entire margin, translucent, rod shaped and Gram negative colonized the roots, leaves and stems of *L. diversifolia*, roots and leaves of *C. Calothyrsus* and leaves of *S. sesban*. Filamentous white colonies with irregular margins, opaque, rod shaped and Gram negative were present in the leaves of all the plants. Cream colonies that were raised with entire margins, opaque and Gram negative bacilli were found to colonize leaves, stem and roots of *S. Sesban, C. Calothyrsus* and stem of *L. diversifolia.* White colonies, raised with undulated margins, opaque, rod shaped which stained purple, were recovered from the three parts of *C. Calothyrsus.* Raised yellow light colonies with entire margin, opaque in opacity, rods in shape and Gram negative were found in the leaves and stems of *L. diversifolia* and *C. Calothyrsus* while white colonies that are flat on the media surface with entire margins, translucent, cocci in shape and Gram positive were recovered from stems of *S. sesban* and leaves of *L. diversifolia*.

**Table 2:** Morphological characteristics of bacterial isolates from *C. calothyrsus*, *L. diversifolia* and *S. sesban*

Bacterial Isolates	Colony Characteristics				*C. calothyrsus*			*S. sesban*			*L. diversifolia*				
	colour	elevation	margin	opacity	L	S	R	L	S	R	L	S	R	G. stain	shape
***BLL4, BLS5***	White	flat	entire	translucent	–	–	–	–	+		+			+ ve	cocci
***BRC1, BSL1, BSC1, BLS1, BSS1, BRS1, BLC1,***	Yellow	raised	entire	opaque	+	+	–	+	+	+	+	+	+	− ve	cocci
***BSL3, BLL3,BSS2***	White	raised	entire	opaque	+	+	+	+	+	+	+	+	+	− ve	cocci
***BLC3, BRL3, BLS2***	White	flat	entire	Translucent	+	–	+	+	-	-	+	+	+	− ve	bacilli
***BLC4***	White	filamentous	irregular	opaque	+	–	–	+	–	–	+	–	–	− ve	bacilli
***BLS3, BRC, BSS3, BLC5, BRS3, BSC2, 3***	Cream	raised	entire	opaque	+	+	+	+	+	+	–	+	–	− ve	bacilli
***BRC5, BSC5, BLC6***	White	raised	undulated	opaque	+	+	–	–	–	–	–	–	–	+ ve	bacilli
***BLL6, BSC3***	Light yellow	raised	entire	opaque	–	+	-	–	–	–	+	–	–	− ve	bacilli

Key: L- leaves, S-stem, R-roots, + -present, -absent

### Molecular characterization

A total of 27 pure bacterial isolates were successfully amplified and sequenced using 16S rRNA primers. Analysis of 16S rRNA gene sequence revealed closely related bacterial species belonging to 8 genera. Members of the genus *Bacillus* (33.3%) dominated the isolates followed by genus *Staphylococcus* (22.2%), *Alcaligens* and *Pantoea,* at 11.2% each. Genus *Xanthomonas* and *Sphingomonas* each had 2 isolates while other genera with 1 isolate each were *Pseudomonas,* and *Acinetobacter*. Isolates (BLS1, BLS2, BLS3, BRC3, BRC5, BSS3, BLS5, BRS1, BSC2, BSC5, BSL3 and BRL5) constituted 66% of the isolates and belonged to phylum proteobacteria. A total of 44% of the isolates (BLL4, BLL6, BSL1, BLC4, BLC5, BLC6, BSS1, BSS2, BRS3, BLC1, BLC3, BLL5, BRC1, BRC3 and BSC1) belonged to phylum firmicutes (Table 3). Most of the isolates had sequences with ˃97.00% identity match with gene bank sequences except for isolate BRS1 and BSC2 whose match identity was 94.6% and 95.9% respectively. Sequences of the isolates were registered in the NCBI Bankit with accession numbers ranging from MW251519.1 to MW251545.1 (Table 3).

**Table 3:** Maximum nucleotide identity matches of bacterial isolates based on 16S rRNA sequences

NO	Isolate ID (Gene Bank Accession)	% Match identity	Close identity to isolate from gene bank	Gene Bank Accession	Source of gene bank close identity
1	BLL4 (MW251519.1)	99.86	*Staphylococcus pasteuri*	KP267845.1	Caesalpinia plant
2	BLL6 (MW251521.1)	99.48	*Staphylococcus epidermidis*	OP811611.1	Vanilla plant
3	BSL1 (MW251522.1)	99.93	*Staphylococcus warneri*	MN181250.1	Tomato root
4	BLC4 (MW251536.1)	100	*Staphylococcus epidermidis*	MN068816.1	Fruits
5	BLC5 (MW251537.1)	99.49	*Staphylococcus sp*	MH191107.1	Syngonium plant
6	BLC6 (MW251538.1)	100	*Staphylococcus pasteuri*	MK308607.1	Coral soils
7	BSS1 (MW251529.1)	99.78	*Bacillus tequilensis*	JN700073.1	Panax plant
8	BSS2 (MW251530.1)	99.85	*Bacillus sp.*	MZ310799.1	Rice
9	BRS3 (MW251533.1)	99.29	*Bacillus toyonensis*	MN543844.1	Rice
10	BLC1 (MW251534.1)	100	*Bacillus altitudinis*	OQ221514.1	Banana
11	BLC3 (MW251535.1)	98.96	*Bacillus toyonensis*	OP811841.1	Vanilla plant
12	BLL5 (MW251520.1)	99.35	*Bacillus toyonensis*	MN543844.1	Rice
13	BSC1 (MW251539.1)	99.08	*Bacillus toyonensis*	MN543844.1	Rice
14	BSC3 (MW251541.1)	100	*Bacillus cereus*	OR514229.1	Rabi crops
16	BRC1 (MW251543.1)	99.25	*Bacillus toyonensis*	OR514203.1	Rabi crops
16	BLS1 (MW251525.1)	92.70	*Alcaligenes faecalis*	MN889404.1	Rice
17	BLS2 (MW251526.1)	87.08	*Alcaligenes faecalis*	MN889375.1	Rice
18	BLS3 (MW251527.1)	98.91	*Alcaligenes faecalis*	MT378145.1	Brassica
19	BRC3 (MW251544.1)	99.29	*Sphingomonas echinoides*	MW021486.1	Sugarcane
20	BRC5 (MW251545.1)	99.21	*Sphingomonas echinoides*	MW021486.1	Sugarcane
21	BSS3 (MW251531.1)	98.19	*Acinetobacter johnsonii*	OL322701.1	Potato
22	BLS5 (MW251528.1)	97.81	*Pantoea agglomerans*	OP595673.1	Bleu berry
23	BRS1 (MW251532.1)	94.64	*Pantoea agglomerans*	OP102642.1	Selex root
24	BSC2 (MW251540.1)	95.9	*Pantoea agglomerans*	OP595673.1	Bleu berry
25	BSC5 (MW251542.1)	99.03	*Pseudomonas plecoglossicida*	MK519202.1	Magnolia
26	BSL3 (MW251523.1)	99.93	*X.campestris pv. campestris*	MT645246.1	Beans
27	BRL3 (MW251524.1)	99.85	*X.campestris pv. campestris*	MT645246.1	Beans

### Phylogenic analysis

**Fig. 1:**
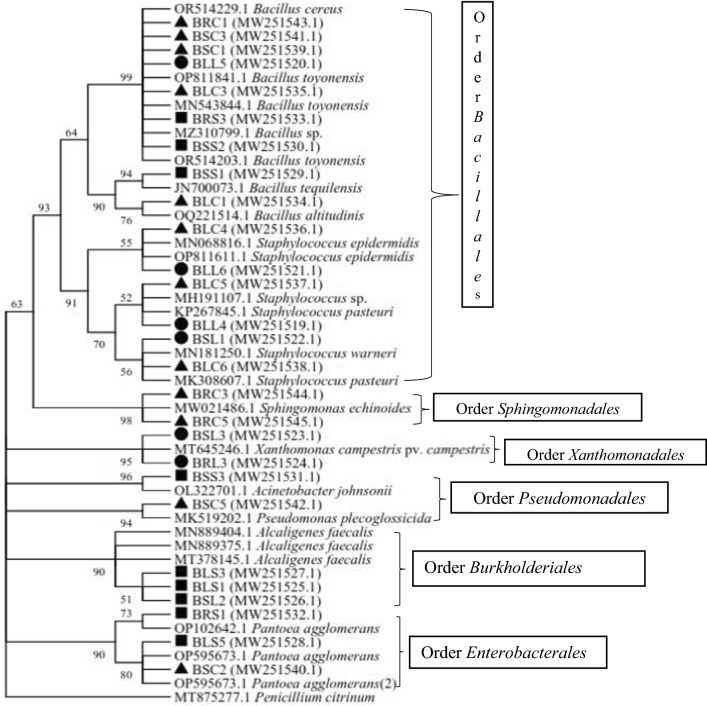
Neighbour joining phylogenetic tree of bacterial isolates of *S. sesban, C. calothyrsus,* and *L. diversifolia* isolates. Key: ●- *L. diversifolia* isolates, ■-*S. sesban* isolates*,* ▲-*C. calothyrsus* isolates

The phylogenetic tree constructed using the isolate sequences and those retrieved from the gene bank clustered the isolates into 6 clades (Fig. 1). The clades represented orders which included *Bacillales, Xanthomonadales Sphingomonadeles, Burkholderiales, Pseudomonodales* and *Enterobacterales*. Order *bacillales* comprised of isolates belonging to two genera; *Bacillus* and *Staphylococcus.* Genus *Bacillus* had 9 sequences compared to Staphylococcus that had 6 sequences clustering at 99% and 91% bootstrap respectively. Isolates in the order *Bacilllales* colonized all 3 plants (*S. Sesban,C. Calothyrsus* and *L. Diversifolia*)*.* Order *Xanthomonadales* and *Sphingomonadeles* comprised of 2 isolates with bootstrap support of 95% and 98%. Order *Enterobacterales* had isolates belonging to genus *Pantoea* which clustered at 90% bootstrap support. Order *pseudomonodales* comprised of genus *Pseudomonas* and *Acinetobacter* while genus *Alcalgenes* belonged to order *Burkholderiales*. Bacterial endophytes in the order *Pseudomonodale*s were isolated from *S. Sesban* and *C. Calothyrsus* while endophytes in the order Burkholderiales were recovered from *L. diversifolia* and *C. calothyrsus.*

## Discussion

The recovery of 27 pure bacterial isolates in this study is an indication of occurrence of diverse endophytes in different parts of *S. sesban*, *C. calothyrsus*, and *L. diversifolia*. Similar results were reported by other researchers (Benjelloun et al. 2019; Bind and Nema 2019) who isolated endophytic bacteria from Chickpea plants and pigeon pea using the same protocol. The presence of higher bacterial isolates in the leaves compared to the other plant parts could be attributed to the availability of nutrients due to photosynthesis. Chowdhary and Kaushik (2015) and Katoch and Pull (2017) reported that leaves have a high diversity of bacterial endophytes than any other plant part which was the case in this study. Bacterial endophytes often colonize the intercellular spaces of the plant parts because these areas are endowed with an abundance of amino acids, carbohydrates and inorganic nutrients (Kandel et al. 2017; Katoch and Pull, 2017) especially the leaves where photosynthesis takes place. Bacterial endophytes recovered from roots, leaves and stems of *L. diversifolia C. calothyrsus* and *S. sesban* exhibited varied morphological features based on elevation, colour, opacity, shape, opacity and Gram staining (Table 2). Bacterial endophytes with different phenotypic characteristics have also been isolated from Soybean (*Glycine max*) (Nhu et al. 2017). Morphological variation in the colonies of bacterial isolates could be due to the ability of different bacterial species to metabolize different constituents of culture media for colonies to have different shades, shapes and elevations.

Bacterial endophytes exhibit wide variations in their phenotypic characteristics even when they are isolated from the same plant tissue, organ or plant species (Nhu et al. 2017; Sondang et al. 2019). As reported (Padder et al. 2017; Sinha et al. 2017), bacteria synthesize pigments as secondary metabolites by utilizing different nutrients in the media hence the variation in colony colour. Pigments protect bacterial cells from toxicity that results from exposure to visible and ultraviolet light rays which could have brought about variation in pigmentation amongst the bacterial isolates. Bacterial isolates were divided into two groups based on the Gram’s reaction and cell shape as Gram negative bacilli and cocci, Gram positive bacilli and cocci. These results are in line with the report of Bhagya et al. (2019) that the legume Green gram (*Vigna radiata L.*) is colonized by both Gram positive cells and Gram negative cells of bacterial endophytes*.* The variation in colour of bacterial cells after staining is due to the difference in the structural composition of their cell walls. The cell wall of Gram-negative bacteria has a lipid layer called lipopolysaccharide that dissolves when treated with alcohol hence losing the primary stain crystal violet and taking up secondary stain to appear red. Cell walls of Gram positive bacteria contain teichoic acid and thick peptidoglycan layers that retain the primary stain crystal violet on decolourization hence appearing purple (Padder et al. 2017).

Researchers (Padder et al. 2017; Singh et al. 2022; Sinha et al. 2017) have used phenotypic features to characterize bacterial endophytes but they are inadequate for complete identification because of the existence of intermediate forms within a subgroup. Conclusive identification of bacteria requires polyphasic taxonomic approach that puts emphasis on the use of classical methods in combination with modern genetic/molecular techniques (Maulani et al. 2019). Based on the 16S rRNA gene sequence, the majority of the isolates belonged to the genus *Bacillus* and *Staphylococcus.* This may be due to their ability to metabolize available nutrients in the media for growth and at such temperatures as compared to other genera which may not. Moreover, members of these genera do not require enrichment of the media for them to grow. Bacterial endophytes belonging to the genus *Bacilli* enable the host plant to tolerate biotic and abiotic stress (Ek-Ramos et al. 2019). This is achieved by stimulation of immune response, niche competition, and metabolism of phenylpropanoid to produce plant defence through structural support and activation of survival molecule. Brígido et al. (2019) reported similar results during the identification of bacterial endophytes of Chickpea (*Cicer arietinum* L.). Leguminous plants harbour the majority of bacteria belonging to genus *Bacilli* and *Pseudomonas* because of their symbiotic association. Members of the genus *Bacilli* such as *Bacillus amy-loliquefaciens* have been reported to be responsible for the solubilization of zinc, potassium and phosphorous. They are also involved in the production of plant hormone (IAA), nitrogen fixation and synthesis of bio-control agents (Rana et al. 2020) hence their dominance as endophytes of *S. sesban, C. calothyrsus* and *L. diversifolia.*

Phylogenetic analysis of the isolates clustered them into six orders each supported by ˃90% bootstrap with the majority coming from the phylum Proteobacteria. Bacterial endophytes that clustered together in any given order had high similarity in gene structure and nucleotide arrangement enabling their sequences to align close to each other during analysis (Horiike, 2016; Munjal et al. 2018). These findings concur with other reports (Chimwamurombe et al. 2016) which indicated that endophytic bacteria in leguminous plants are dominated by members of phylum Proteobacteria while a few belong to phylum Firmicutes. Diverse genera of bacteria belonging to phylum proteobacteria were isolated in high numbers probably because most of them are culture dependent and do not require special nutrients to grow. Similarly, they are found as endophytes probably because they have evolved different strategies of overcoming plant defence mechanisms to gain entry and systemically move and lodge into different parts of the host plant. Once inside, they improve plant nutrient uptake and stimulate the synthesis of growth promoting as well as stress tolerance hormones. (Zhang et al. 2019). Endophytic bacteria also synthesize secondary metabolites with antimicrobial and anti-insect activities thus enabling the host plant to resist pathogenic attack (Elmagzob et al. 2019; Zhang et al. 2019).

In this study, bacterial endophytes of the genus *Staphylococcus, Bacillus,* and *Alcaligens* were isolated from more than one plant species and plant organ while some were specific to the plant species and organ of colonization. Colonization of more than one plant species could be because the plants belong to the family leguminosaea and secrete exudates with similar nutritional and chemical composition that attracted similar bacterial endophytes. Bacterial endophytes tend to disregard the theory of host specificity thereby becoming naturally promiscuous to interact with different host plants which supports the findings of our study (Card et al. 2016; Tidke et al. 2017). On the other hand, members of genus *Acinetobacteria, Pantoea and Alcaligenes* were specific to the plant and organ of origin. Different plants and organs have varied chemical compositions due to genetic variability that determines the selection and preference of colonizing bacterial endophytes which could be the case in this study. The presence of different bioactive compounds in different plant species and organs dictates the species of bacteria that colonize as endophytes (Maggini et al. 2019). Some of the bioactive compounds that control and dictate endophyte colonization include alkenes, acid derivatives, alkamides, polysaccharides and caffeine. Endophytic bacteria are attracted to their host rhizosphere by exudates rich in different phenolic compounds, amino acids and sugars before penetrating to lodge within the plant (Iyer et al. 2017; Liu et al. 2017; Maggini et al. 2019). Once they are in the rhizosphere, they use different mechanisms to gain entry into the host plant where they will spend either part or whole of their lifecycle.as mutualistic endophytes. In overall, all the isolates separated into Gram positive and Gram negative based on Gram staining and bacilli and cocci based on cell shape. BLAST analysis of the 16S rRNA gene of the isolates revealed eight genera dominated by the genus *Bacillus*. The genera belonged to two phyla with the dominant phylum being proteobacteria. This study therefore demonstrated high diversity of bacterial endophytes from the three leguminous plants.

## Data Availability

The data used in writing the results of this study are part of this article.

## Abbreviations

ZR: Zymo Research
DNA: Deoxyribonucleic acid
SYBR: Synergy Brands
DMSO: Dimethyl sulfoxide
rRNA: Ribosomal ribonucleic acid
dNTP: Deoxynucleoside triphosphate
Taq: Thermus aquaticus
TAE: Tris base, acetic acid and EDTA
NCBI: National Centre for Biotechnology Information database
BLAST: Basic Local Alignment Search Tool
MEGA: Molecular Evolutionary Genetic Analysis
